# Measles resurgence in England: challenges in maintaining elimination, 2023 to 2024

**DOI:** 10.2807/1560-7917.ES.2026.31.45.2600124

**Published:** 2026-11-12

**Authors:** Erjola Hani, Tishya Venkatraman, Julia Stowe, Rebecca Cordery, Mary Ramsay, Vanessa Saliba

**Affiliations:** 1 Immunisation and Vaccine-Preventable Diseases Division, UK Health Security Agency (UKHSA), London, United Kingdom

**Keywords:** Measles, Measles resurgence, Measles elimination, MMR vaccine coverage, Disease surveillance, Epidemiology, Hospitalisation, Health inequalities, United Kingdom

## Abstract

**BACKGROUND:**

Between October 2023 and September 2024, England experienced the largest measles outbreak in decades.

**AIM:**

We describe the epidemiology of the outbreak, burden on secondary care services and the factors that led to this resurgence.

**METHODS:**

The UK Health Security Agency (UKHSA) conducts enhanced surveillance of all suspected measles cases in England. A measles line list was established using routinely collected notification, laboratory, demographic and vaccination information. Laboratory-confirmed cases were defined using serology and PCR. Incidence rates were calculated using mid-2024 population estimates. Severity was assessed through linkage with emergency department attendances, hospital admissions and length of stay.

**RESULTS:**

Most of the 2,821 laboratory-confirmed cases resided in London (n = 1,283; 45.5%) and in the West Midlands (n = 710; 25.2%). Incidence was highest in London (14.1/100,000 population). Children under 10 years represented 58.6% (n = 1,653) of cases. Most cases (n = 2,609; 92.5%) were not vaccinated, and 63.0% (n = 1,776) lived in areas belonging to the two most deprived quintiles. Of the 2,788 cases that could be linked to hospitalisation data, 76.3% (n = 2,127) attended emergency care and 38.2% (n = 1,066) were admitted. Median length of stay was 2.0 days, seven cases received intensive care; one death was reported. Admissions were highest among infants aged < 1 year (51.6%, 165/320) and adults ≥ 30 years (47.2%, 195/413).

**CONCLUSION:**

This outbreak reflects a shift towards endemic measles transmission in areas of high deprivation and low vaccine uptake in England. It underscores the importance of achieving 95% uptake with measles vaccine in all areas and communities to achieve and maintain elimination.

**Figure:**
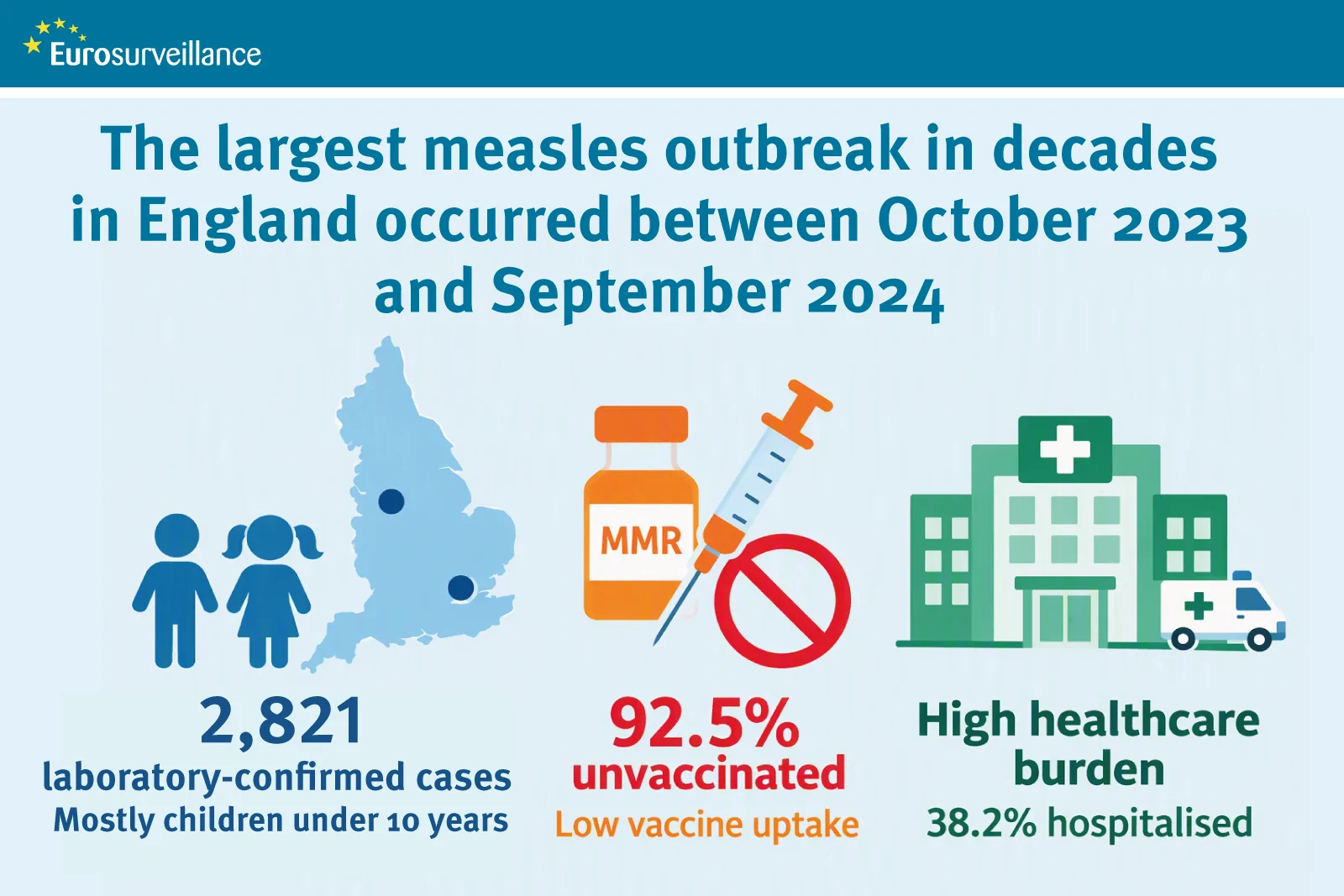

**Key public health message**

**What did you want to address in this study and why?**

After the COVID-19 pandemic, England saw the largest measles outbreak in decades. We wanted to explain why this happened, describe the communities affected and the impact on health services. We also wanted to show how surveillance informed actions to control the outbreak and what led to the World Health Organization (WHO) declaring that measles was once again circulating in the UK.

**What have we learnt from this study?**

Between October 2023 and September 2024, 2,821 persons were laboratory-confirmed with measles. Almost 60% were unvaccinated children under the age of 10 years. The majority lived in the poorest areas of London and Birmingham. Approximately 76% of the cases attended an emergency department and almost half of them stayed in hospital, although most only for one night.

**What are the implications of your findings for public health?**

Routinely collected disease data can be very useful to track where measles is spreading and inform actions to control outbreaks in affected areas and communities. To achieve and sustain measles elimination, health system partners in the United Kingdom need to work together to improve vaccine uptake in children under 5 years of age, especially in the poorest areas and catch-up older children and adults who missed out.

## Background

Measles virus is a highly contagious airborne virus; one individual can infect nine of ten non-immune close contacts. Measles infection can lead to severe complications and death. From 2000 to 2023, measles vaccination averted over 60 million deaths globally [1]. A measles-containing vaccine was introduced into the United Kingdom (UK) routine childhood immunisation schedule in 1968, with a single-dose measles–mumps–rubella (MMR) vaccine introduced in 1988, and two-dose schedule introduced in 1996 [2]. Two doses of the MMR vaccine have been offered routinely at 12 months, and 3 years and 4 months of age [2]. From 1 January 2026, due to the introduction of the varicella programme, the vaccine in use for the routine programme was changed to the combined MMR and varicella (MMRV) vaccine, and the second dose was brought forward to 18 months of age with the aim of improving uptake [3,4].

Following the introduction of the two-dose schedule and high vaccine uptake, endemic measles transmission in the UK was interrupted [2]. In 1998, a discredited paper falsely linking the MMR vaccine to autism caused national MMR vaccination coverage to fall below 80%, increasing the number of susceptible children [2,5,6]. From 2000 to 2013, several measles outbreaks occurred, largely among children who had missed vaccination during this period. Targeted catch-up campaigns, particularly the 2013 MMR campaign for 10–16-year-olds, helped halt transmission and re-establish control [7,8]. By the end of 2014, the UK had interrupted endemic measles transmission for the first time in 13 years [9,10]. Subsequently, the World Health Organization (WHO) confirmed that the UK had eliminated measles in 2016 [9,11].

The WHO recommends 95% coverage with two doses of a vaccine containing measles virus to achieve and sustain measles elimination [1]. In England, coverage of MMR vaccine dose one, evaluated at age of 5 years, reached 95% for the first time in 2016/17; however, coverage declined thereafter [12]. Between 2016 and 2021, many measles outbreaks were seeded across the UK following importations from large outbreaks across Europe [13], and by 2018, the UK had re-established measles transmission [9,11]. Some outbreaks disproportionately affected undervaccinated communities, including people from minority religious groups, Roma and Traveller communities, and recent migrants [14-20]. Measles transmission was halted during the COVID-19 pandemic due to public health measures such as travel restrictions and social distancing. As a result, the UK regained measles elimination status in 2021 [9,11].

In 2023/2024, MMR vaccine coverage reached its lowest levels within a decade, with only 88.9% of children receiving their first MMR dose by 24 months, and 91.9% by 5 years [12]. Analyses conducted by UK Health Security Agency (UKHSA) have highlighted that population immunity levels in the UK are below those required to interrupt measles transmission in many birth cohorts, with London and other inner-city areas particularly at risk of large, sustained outbreaks [21].

In 2023, in the wake of a decade long decline in routine vaccine uptake [22], there was a global resurgence of measles, and the WHO European Region reported a 30-fold increase in measles cases, attributed to declining childhood vaccination rates [23,24]. This same pattern was observed in the UK [25,26], with a large outbreak commencing in the final quarter of 2023, initially concentrated in the West Midlands, with transmission spreading to London and then to all other regions in 2024 [25-27].

This paper describes the epidemiology of the largest measles outbreak in England in over two decades [28,29]. We used enhanced surveillance data linked to other datasets to characterise measles outbreaks in England, demonstrating the value of this approach to track importations and clusters as they emerged, characterise communities affected to inform interventions and assess impact on secondary care services.

## Methods

### Measles surveillance, case finding and laboratory testing

In England, measles is a notifiable disease; suspected cases should be reported by clinicians to local UKHSA Health Protection Teams (HPTs) and recorded in the case incident management system (CIMS) [30]. In accordance with national measles guidance, clinically suspected measles cases undergo risk assessment and are sent an oral fluid kit (OFK) for self-swabbing and return to the UKHSA Virus Reference Department (VRD) for serology (IgM and IgG) and molecular (PCR) testing [31]. Also, UKHSA requests that locally tested positive diagnostic samples are forwarded to VRD for confirmatory testing [31]; any sample testing positive on PCR can then undergo genotyping and sequencing.

Measles testing data from VRD were accessed via UKHSA’s Modular Open Laboratory Information System (MOLIS), while all measles-positive IgM and PCR results from all English laboratories were reported to the Second-Generation Surveillance System (SGSS).

Data from CIMS, MOLIS and SGSS are extracted and linked weekly using National Health Service (NHS) numbers and other patient identifiers to maintain a national measles line list for England; including all laboratory-confirmed, discarded and suspected cases under investigation. The data flow is presented in Supplementary Figure S1. Cases are classified as discarded if they test negative by serology or molecular testing at the VRD or local laboratories or are classified as discarded in CIMS (Box).

Box **Case definition of laboratory-confirmed measles cases, England**

**Inclusion criteria:**

• Individuals with measles IgM antibodies detected in blood or oral fluid or confirmed wild type measles RNA in any clinical specimen, reported through MOLIS.

• Individuals with wildtype measles RNA or IgM antibodies detected in samples tested locally, reported via SGSS.

• Individuals reported as laboratory-confirmed by HPTs on CIMS, even if no testing information is reported in SGSS/MOLIS. These cases are followed up with the HPTs to confirm the source of local laboratory confirmation.

**Exclusion criteria:**

• Individuals who had a positive result from a local laboratory but were discarded after a negative result from a sample received for processing at VRD. These cases were reviewed by a clinician (clinical symptoms, epidemiological links and laboratory data) to determine inclusion or exclusion.

• Individuals with laboratory-confirmed measles who reported vaccination within the 14 days before symptom onset underwent a review by a clinician (vaccine dates vs symptom onset dates, epi-link to a confirmed case and offer of vaccination as post exposure prophylaxis) and in keeping with WHO guidance were classified as vaccine-associated and excluded.

• Individuals who were confirmed to have vaccine-virus detected on genotyping were also excluded.

CIMS: case incident management system of UKHSA; HPT: UKHSA Health Protection Team; MOLIS: Modular Open Laboratory Information System of UKHSA; SGSS: Second-Generation Surveillance System; UKHSA: United Kingdom Health Security Agency; VRD: UKHSA Virus Reference Department; WHO: World Health Organization.

### Enhanced national measles line list

The date of onset of rash or other symptoms relevant for measles was derived from the clinical case record in CIMS. Where missing, the date of the record creation in CIMS or the date of sample collection were used as proxies. Age in years was calculated from the difference between date of birth and date of onset. This analysis included cases with symptom onset between 1 October 2023 and 30 September 2024.

The national measles line list was enhanced, through linkage using NHS number, with the Immunisation Information System (IIS), a source of primary care MMR records in England provided by NHS England. Cases were classified as vaccinated (1 or 2 doses) if vaccination occurred at least 14 days before symptom onset. Any MMR doses given before 12 months of age were discounted. The total number of doses was determined using intervals of more than 28 days between doses.

Patient ethnicity was derived from the IIS dataset, with missing data supplemented through linkage to Hospital Episode Statistics and the Maternity Services Data Set. Following enrichment, ethnicity data remained unavailable for 5.3% (150/2,821) of cases. Ethnicity was categorised into broader ethnic groups for analysis.

Index of Multiple Deprivation (IMD) quintiles were derived for each case, ranked from the most deprived area (rank 1) to the least deprived area (rank 5). The IMD quintile could not be calculated for 0.6% (17/2,821) cases due to missing postcode. Office for National Statistics (ONS) 2024 geographical lookups were linked to cases’ residential postcodes to identify regional and lower-tier local authority (LTLA) geographies. To identify deaths, we linked the line list to NHS demographic records that includes date of death. Information on foreign travel is collected as part of risk assessments conducted by HPTs. Cases are individually reviewed and classified as imported if they returned to England within 21 days of symptom onset [31].

### Analysis

Incidence rates were calculated using ONS 2024 mid-year population estimates aggregated at LTLA level [32].

The enhanced national measles line list was linked weekly via NHS number to the Emergency Care Data Set (ECDS) and Secondary Uses Service (SUS). An NHS number was missing for 1.2% (33/2,821) of confirmed cases; these cases were excluded from hospitalisation analysis. The ECDS was used to identify Emergency Department (ED) attendance from 5 days before to 10 days after symptom onset. Only one attendance per individual was included. Onward admission was also identified through ECDS. Additional hospital admissions from 5 days before to 10 days after symptom onset were identified with SUS. Data on ED attendance and hospital admission were stratified by age to calculate proportions, using measles cases with recorded NHS numbers as denominators.

Length of stay (LOS) was calculated from SUS using the difference between admission and discharge dates, but LOS could not be calculated for cases which were flagged as admitted in ECDS (129/1,066; 12.1%), due to discharge date fields not being available. We stratified LOS by age as a proxy for disease severity. We identified ICU admission in SUS using treatment function codes ‘192’ or ‘194’, and procedure codes ‘%E85%’, ‘%E89%’ and ‘%X58%’.

## Results

### Testing

Between 1 October 2023 and 30 September 2024, 15,587 suspected measles cases were reported to UKHSA. As per guidance, all were sent an OFK, with some also offered local testing following presentation to healthcare settings. Of the suspected cases, 42.9% (n = 6,684) returned an OFK to the VRD, a further 2,619 individuals tested through local laboratories, increasing the proportion tested to 59.7%. As reporting of negative measles results by local laboratories is not mandatory, this is likely an underestimate. This paper describes the 2,821 laboratory-confirmed cases identified during the outbreak period, corresponding to an incidence of 4.8 cases per 100,000 population.

### Regional distribution

We observed a rapid increase in measles cases in late 2023, driven by an outbreak in Birmingham (West Midlands) which escalated rapidly and seeded all other regions in England. In 2024, activity was predominantly concentrated in London, peaking in April and declining from July onwards, coinciding with the summer school holidays (Figure 1). Nonetheless, all regions reported cases during the outbreak; most cases were in London (1,283/2,821; 45.5%), the West Midlands (710/2,821; 25.2%) and the East of England (206/2,821; 7.3%) (Figure 1, Figure 2). However, the incidence rate was highest in London (14.1/100,000), West Midlands (11.5/100,000) and the East Midlands (3.8/100,000) (Figure 2). The most affected LTLAs across England during the outbreak were Lambeth (55.9/100,000; n = 177), Wandsworth (45.3/100,000; n = 153), Birmingham (40.5/100,000; n = 480), Harrow (39.2/100,000; n = 106) and Middlesborough (34.6/100,000; n = 54) (Figure 2).

**Figure 1:**
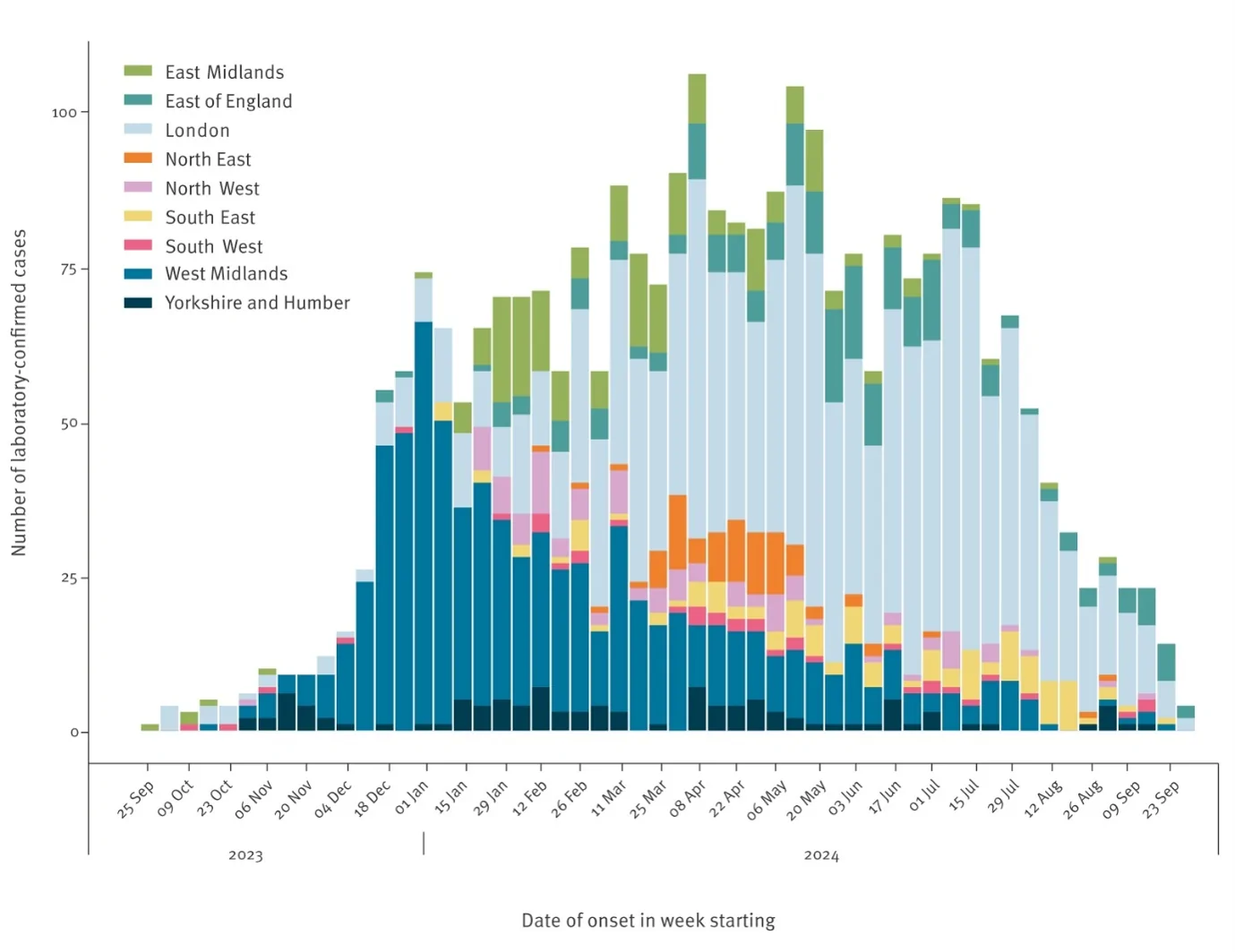
Epidemiological curve showing the weekly number of laboratory-confirmed measles cases, by region of residence, England, 1 October 2023–30 September 2024 (n = 2,821)

**Figure 2:**
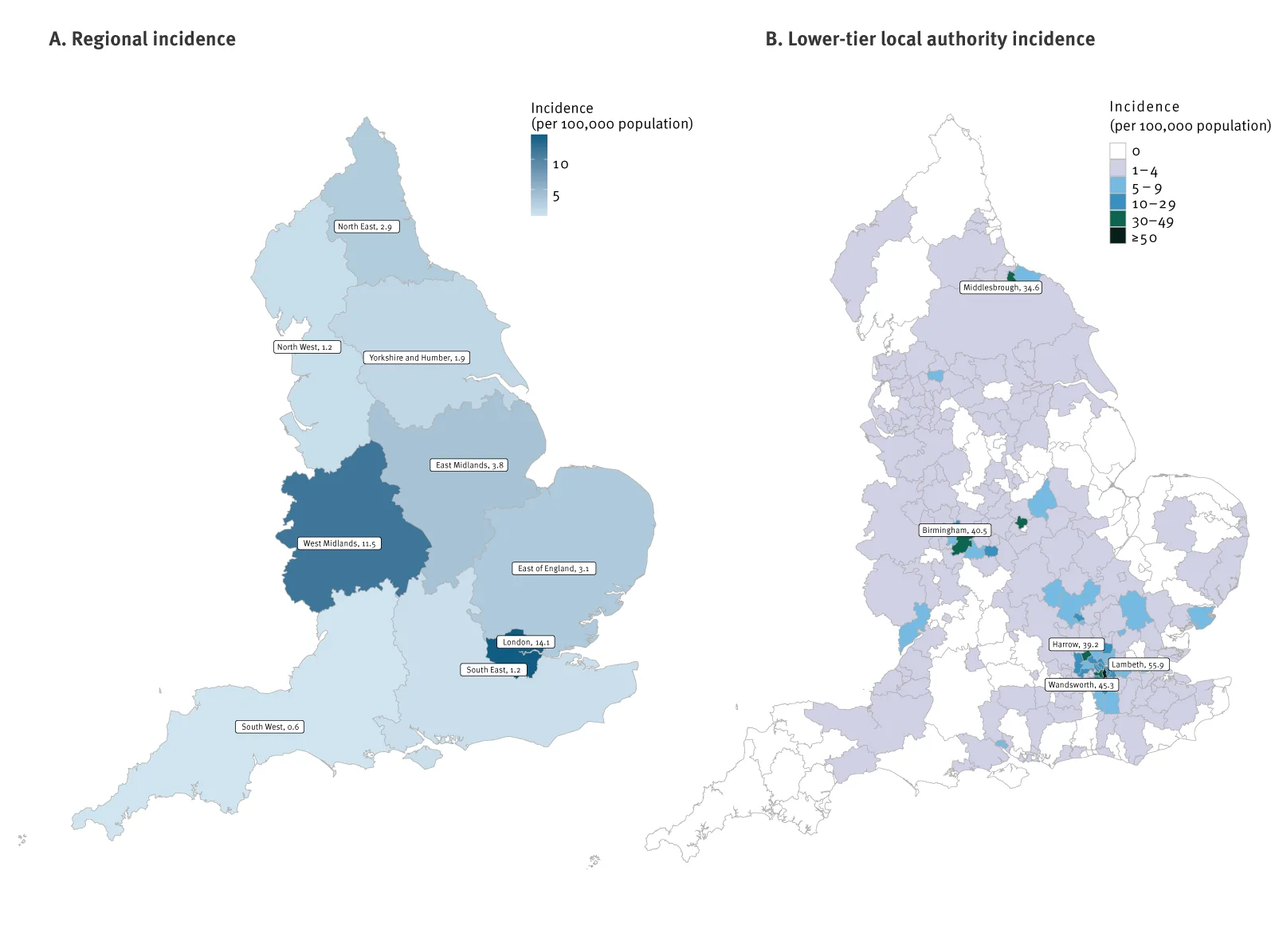
Geographical distribution of measles incidence, England, 1 October 2023–30 September 2024 (n = 2,821)

LTLA: lower-tier local authority; ONS: Office for National Statistics.

Panel A. Regional incidence of confirmed measles cases per 100,000 population, calculated using ONS mid-2024 population estimates. Regions are shaded on a continuous colour scale, with darker shading indicating higher incidence.

Panel B. LTLA incidence of confirmed measles cases for the same period, calculated using ONS mid-2024 population estimates. LTLAs are displayed without incidence shading for clarity. The five LTLAs with the highest incidence are labelled with their incidence values (per 100,000 population).

### Age distribution

Most cases were in children aged under 10 years (1,653/2,821; 58.6%), with outbreaks frequently linked to nursery and primary school settings. Infants (children aged under 1 year), who are not routinely eligible for MMR vaccination, accounted for 11.7% (329/2,821) of all cases (Table 1). Around a third of cases were reported in persons aged 15 years and over (880/2,821). Most cases were unvaccinated, with 3.8% (108/2,821) vaccinated with one dose of MMR, and 3.7% (104/2,821) vaccinated with two doses.

**Table 1:** Demographic characteristics of laboratory-confirmed measles cases, by region, England, 1 October 2023–30 September 2024 (n = 2,821)

Characteristic	Total		NHS region																		
			East Midlands (n = 190)		East of England (n = 206)		London (n = 1,283)		North East (n = 79)		North West (n = 96)		South East (n = 113)		South West (n = 38)		West Midlands (n = 710)			Yorkshire and Humber (n = 106)	
	n	%	n	%	n	%	n	%	n	%	n	%	n	%	n	%	n	%	n		%
Age group (years)																					
< 1	329	11.7	9	4.7	23	11.2	148	11.5	15	19.0	11	11.5	15	13.3	5	13.2	91	12.8	12		11.3
1–4	703	24.9	50	26.3	59	28.6	319	24.9	16	20.3	20	20.8	13	11.5	7	18.4	190	26.8	29		27.4
5–9	621	22.0	54	28.4	47	22.8	325	25.3	15	19.0	12	12.5	10	8.8	5	13.2	133	18.7	20		18.9
10–14	288	10.2	29	15.3	19	9.2	111	8.7	12	15.2	7	7.3	10	8.8	0	0.0	96	13.5	4		3.8
15–19	167	5.9	15	7.9	15	7.3	54	4.2	4	5.1	8	8.3	6	5.3	4	10.5	60	8.5	1		0.9
20–29	292	10.4	15	7.9	18	8.7	134	10.4	3	3.8	13	13.5	18	15.9	7	18.4	63	8.9	21		19.8
≥ 30	421	14.9	18	9.5	25	12.1	192	15.0	14	17.7	25	26.0	41	36.3	10	26.3	77	10.8	19		17.9
Sex																					
Female	1,299	46.0	88	46.3	113	54.9	583	45.4	31	39.2	42	43.8	59	52.2	19	50.0	312	43.9	52		49.1
Male	1,483	52.6	98	51.6	91	44.2	676	52.7	47	59.5	54	56.3	54	47.8	19	50.0	391	55.1	53		50.0
Unknown	39	1.4	4	2.1	2	1.0	24	1.9	1	1.3	0	0.0	0	0.0	0	0.0	7	1.0	1		0.9
Vaccination status																					
1 dose	108	3.8	6	2.6	15	7.3	36	2.8	3	3.8	6	6.3	1	0.9	3	7.9	36	5.1	3		2.8
2 doses	104	3.7	6	3.2	7	3.4	33	2.6	2	2.5	8	8.3	9	8.0	2	5.3	28	3.9	9		8.5
No vaccination recorded	2,609	92.5	179	94.2	184	89.3	1,214	94.6	74	93.7	82	85.4	103	91.2	33	86.8	646	91.0	94		88.7
Foreign travel																					
Imported	155	5.5	14	7.4	14	6.8	56	4.4	1	1.3	10	10.4	16	14.2	12	31.6	19	2.7	13		12.3
Not imported	2,666	94.5	176	92.6	192	93.2	1,227	95.6	78	98.7	86	89.6	97	85.9	26	68.4	691	97.3	93		87.7
Genotype																					
b3	572	20.3	15	7.9	12	5.8	252	19.6	1	1.3	43	44.8	15	13.3	11	28.9	208	29.3	15		14.2
d8	1,597	56.6	140	73.7	164	79.6	831	64.8	60	75.9	33	34.4	83	73.5	21	55.3	203	28.6	62		58.5
Not sequenced	652	23.1	35	18.4	30	14.6	200	15.6	18	22.8	20	20.8	15	13.3	6	15.8	299	42.1	29		27.4
Index of multiple deprivation quintile^a^																					
1	1,057	37.5	52	27.4	36	17.5	285	22.2	56	70.9	47	49.0	12	10.6	8	21.1	501	70.6	60		56.6
2	719	25.5	62	32.6	45	21.8	436	34.0	6	7.6	12	12.5	18	15.9	8	21.1	120	16.9	12		11.3
3	507	18.0	32	16.8	53	25.7	303	23.6	5	6.3	14	14.6	23	20.4	10	26.3	52	7.3	15		14.2
4	344	12.2	33	17.4	41	19.9	187	14.6	5	6.3	15	15.6	29	25.7	5	13.2	21	3.0	8		7.5
5	177	6.3	11	5.8	31	15.0	66	5.1	5	6.3	8	8.3	31	27.4	7	18.4	10	1.4	8		7.5
Unknown	17	0.6	0	0.0	0	0.0	6	0.5	2	2.5	0	0.0	0	0.0	0	0.0	6	0.8	3		2.8
Ethnicity																					
White	1,330	47.1	73	38.4	155	75.2	559	43.6	55	69.6	66	68.8	85	75.2	29	76.3	250	35.2	58		54.7
Black/African/Caribbean/Black British	491	17.4	8	4.3	10	4.9	328	25.6	2	2.5	1	1.0	2	1.8	0	0.0	134	18.9	6		5.7
Asian/Asian British	393	13.9	64	33.7	11	5.3	91	7.1	3	3.8	14	14.6	10	8.8	2	5.3	176	24.8	22		20.8
Mixed/Multiple ethnic groups	267	9.5	19	10.0	14	6.8	149	11.6	5	6.3	6	6.3	8	7.1	1	2.6	60	8.5	5		4.7
Other	190	6.7	17	8.9	4	1.9	102	8.0	5	6.3	6	6.3	4	3.5	1	2.6	44	6.2	7		6.6
Unknown	150	5.3	9	4.7	12	5.8	54	4.2	9	11.4	3	3.1	4	3.5	5	13.2	46	6.5	8		7.5

NHS: National Health Service.

^a^ Based on Index of Multiple Deprivation (IMD) quintiles. The IMD datasets are small area measures of relative deprivation across each of the constituent nations of the United Kingdom. Areas are ranked from the most deprived area (rank 1) to the least deprived area (rank 5).

### Genotyping and importations

Overall, samples from 76.9% (2,169/2,821) of cases were genotyped. The majority were d8 strain (n = 1,597) with the remainder b3 (n = 572) (Table 1). Although both genotypes circulated concurrently in all regions throughout the outbreak period, d8 dominated, particularly in London. The early West Midlands outbreaks were seeded by two importations, a b3 from Pakistan and d8 from Romania. However, there were multiple importations throughout the outbreak, some of which were linked to seeding events that resulted in community transmission. Of the cases, 5.5% (n = 155) were classified as imported (Table 1); of these, around a third were linked to travel to Romania (n = 50).

### Deprivation

A clear pattern of cases concentrated in the most deprived areas emerged early in the outbreak, particularly in the West Midlands. This deprivation gradient persisted throughout the outbreak, across all regions, with 63% (1,776/2,821) of cases resident in the most deprived areas (quintiles 1 and 2) of England (Table 1).

### Hospitalisation and severity

During the outbreak, 76.3% (2,127/2,788) of the laboratory-confirmed cases with an NHS number presented to an ED (Table 2). Among cases attending ED, 46.7% (994/2,127) were subsequently hospitalised, while the remainder (1,133/2,127; 53.3%) were discharged without admission.

**Table 2:** Laboratory-confirmed measles cases attending an emergency department, by age group, England, 1 October 2023–30 September 2024 (n = 2,788)

Age group (years)	Cases (n = 2,788)^a^	Cases attending ED (n = 2,127)		Cases attending ED only (n = 1,133)		Cases admitted to hospital (n = 1,066)^b^	
		N	%	n	%	n	%
< 1	320	273	85.3	121	37.8	165	51.6
1–4	698	557	79.8	308	44.1	271	38.8
5–9	618	447	72.3	299	48.4	162	26.2
10–14	288	205	71.2	128	44.4	84	29.2
15–19	164	126	76.8	73	44.5	54	32.9
20–29	287	226	78.7	97	33.8	135	47.0
≥ 30	413	293	70.9	107	25.9	195	47.2

ED: emergency department.

^a^ Cases without an NHS number were excluded (n = 33) from the total number of confirmed cases (n = 2,821).

^b^ Hospitalised via ED: n = 944; hospitalised via other care routes: n = 72. Ages of measles cases hospitalised via other care routes: < 1 years: n = 13; 1–4 years: n = 22; 5–9 years: n = 14; 10–14 years: n = 7; 15–19 years: n = 1; 20–29 years: n = 6; ≥ 30: n = 9.

Source: Secondary Uses Service dataset; Emergency Care dataset; Enhanced national measles line list.

Overall, 40.6% (1,133/2,788) of laboratory confirmed cases with an NHS number attended ED without subsequent admission. This proportion was highest for the 5–9-year-olds (299/618; 48.4%) and lowest for adults aged over 30 years (107/417; 25.7%) (Table 2).

Of all laboratory-confirmed measles cases with an NHS number, 38.2% (1,066/2,788) were admitted to hospital. Most were admitted from ED, however a small proportion (72/1,066; 6.8%) were hospitalised via other care routes, for example through direct referral by their general practitioner (GP). The proportion hospitalised was highest among infants (165/320, 51.6%) and the lowest among children aged 5–9 years (162/618; 26.2%) (Table 2).

The remaining 21.2% (589/2,788) of all laboratory-confirmed cases with an NHS number had no recorded interaction with a tertiary healthcare setting.

Among the cases flagged as admitted through SUS (n = 937), the median LOS was 2.0 days. The vast majority of cases (794/937; 84.7%) were hospitalised for ≤ 4 days (Figure 3A). Length of hospital stay was highest for cases aged ≥ 30 years (4.0 days), those aged 15–19 years (3.0 days) and infants (2.8 days) (Figure 3B). Among admitted cases, 88.7% (831/937) had an ICD-10 discharge diagnosis recorded, of these, 70.0% (582/831) included a measles code (B05) as a primary or secondary diagnosis. The other, most frequently reported codes were R21 (rash and other nonspecific skin eruption), B34.9 (unspecified viral infection) and J22 (unspecified acute lower respiratory infection).

**Figure 3:**
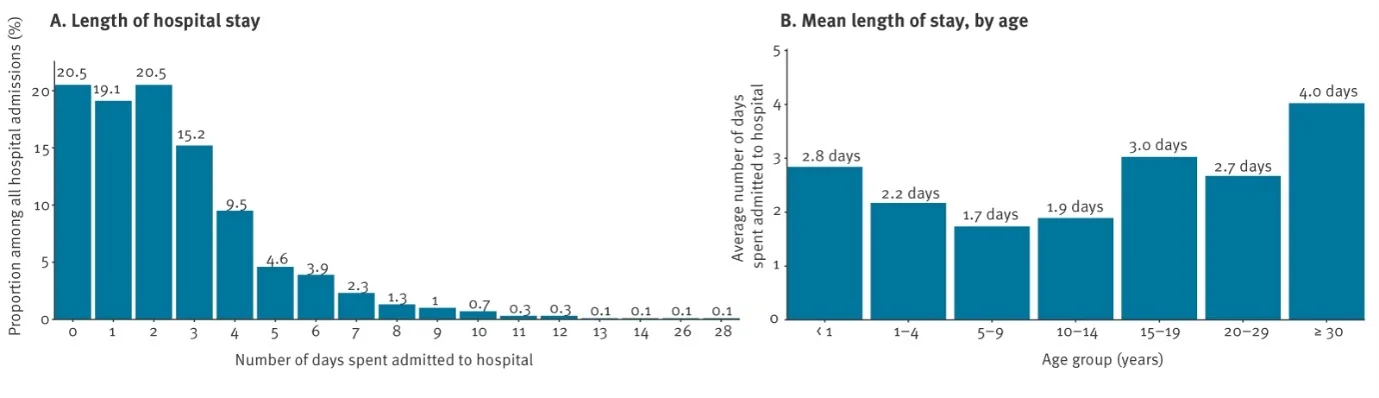
Length of hospital stay among laboratory-confirmed measles cases, England, 1 October 2023–30 September 2024 (n = 937)

Panel A. Three cases (0.3%) had a length of stay over 30 days.

Data sources: Secondary Uses Service (SUS), Enhanced national measles line list.

Overall, seven cases were recorded as having been admitted into an intensive care unit during their hospital admission, of these individuals three had underlying comorbidities. One acute measles death was reported, in a teenager with underlying health conditions.

## Discussion

In 2023–2024, England experienced the largest measles outbreak in two decades, particularly affecting unvaccinated children under the age of 10 years in deprived inner-city areas. Transmission was geographically widespread, with substantial heterogeneity in incidence at regional and more granular local level. Initial importations seeded the outbreaks within highly deprived areas of Birmingham and other parts of the West Midlands. Analysis conducted by the West Midlands field service team identified co-location of undervaccinated communities, with unvaccinated children attending the same nurseries and primary schools, facilitating onward transmission [27]. Our genomic and epidemiological analysis indicated that population movement within England quickly facilitated spread to London and all other regions, leading to community transmission in some areas. Overall, London and the West Midlands were the most affected regions, with noted high-incidence hotspots. Assessing epidemiology at a more granular geographic level enables identification of localised transmission and supports targeted interventions.

A 2023 UKHSA risk assessment predicted a large outbreak in London [21]; however, although nearly half of all cases occurred there, observed case numbers were substantially lower than expected. This may reflect overestimation of population susceptibility due to incomplete capture of vaccination status in routine GP records among older children and adults due to high mobility, inward migration and population turnover [33].

Two-thirds of cases resided in the most deprived areas, consistent with lower MMR uptake [12] and 92.5% of cases were unvaccinated individuals, reinforcing the high effectiveness of measles vaccination in preventing disease [2]. Nonetheless, barriers to service access [34], pandemic-related disruption to routine healthcare services and vaccine hesitancy remain important contributors of declining coverage of childhood immunisations, exacerbating existing immunity gaps and leading to a measles resurgence across Europe [35].

In contrast to pre-pandemic epidemiology in the UK, cases in this outbreak were more broadly distributed across communities and ethnic groups, rather than concentrated in a particular undervaccinated population. Transmission was highest among unvaccinated children under the age of 5 years.

Emergency department attendance and admission rates were high, at around 75% and 38% of laboratory confirmed cases, respectively. This was partly attributable to the age distribution of cases, which was skewed towards young children. Rates were also likely influenced by the high proportion of cases residing in deprived areas, where patterns of healthcare-seeking behaviour may result in greater use of emergency department services as an alternative to GP [36].

Duration of hospitalisation is likely a better proxy for severity than admission alone. Most admissions lasted only 1–2 days which suggests many children were admitted overnight for observation and subsequently discharged. Longer admissions were observed in adults aged >30 years (4.0 days), 15–19-year-olds (3.0 days) and infants (2.8 days), which is consistent with historical data [37]. In line with expected severity, only a small number of ICU admissions and a single death were reported during the outbreak period. Given that all suspected cases consulted a health professional and many required ED visits or admissions, this measles outbreak placed a substantial burden on the NHS.

From January to November 2024, UKHSA convened a national incident to coordinate the multi-agency risk assessment and response at national, regional and local levels. To address this measles resurgence, NHS England launched a national MMR catch-up campaign, initially targeting children aged 12 months to 5 years (November 2023), followed by unvaccinated or partially vaccinated children aged 6–11 years (February 2024), as informed by the epidemiology. The catch-up was extended to individuals aged 12–25 years in London, Greater Manchester and the West Midlands. These formed part of a broader package of measures to improve MMR uptake, including targeted local interventions in outbreak areas such as school-based delivery via School Age Immunisation Services and community outreach clinics [7,38], alongside a phased national childhood immunisation marketing campaign (CHIMMS) [38].

A UKHSA evaluation showed that an estimated 180,000 additional doses were delivered between 31 August 2023 to 30 April 2024 [33], with the greatest impact observed in groups with lower baseline coverage, contributing to a narrowing of inequalities by deprivation and ethnicity [33]. The evaluation concluded that sustained improvements in MMR coverage will require long-term, well-resourced and locally tailored interventions, particularly in populations and areas with the lowest uptake [33]. For example, school entry could be used as a key opportunity for vaccine check and offer; this is consistent with recent European policy emphasis on closing existing immunisation gaps beyond routine infant schedules [35].

A steady decline in cases started in July 2024; however, due to evidence of sustained transmission for a period exceeding 365 days, the UK lost its elimination status [9]. Ongoing transmission into 2025 and 2026 [39] further highlights the importance of higher-resolution approaches to characterise transmission dynamics, including sequencing-based inference and reconstruction of transmission chains from 2023 onwards. Lastly, in the context of a global resurgence of measles, the UK’s high global connectivity and population mobility increases the risk of repeated importations leading to ongoing outbreaks, if immunity gaps are not addressed [40].

One of the main limitations of this study is under-ascertainment of cases. Mild cases may not present to healthcare and are therefore less likely to be notified or tested, whereas severe cases are more likely to present to healthcare, hence be captured, biasing laboratory-confirmed cases towards greater severity. Similarly, notified suspected cases with mild symptoms are less likely to return an OFK leading to underestimation of laboratory confirmed cases.

For the hospitalisation analysis, all laboratory-confirmed cases linked to secondary care datasets within the defined time window were included regardless of admission reason. Nearly 90% of measles cases admitted had an ICD-10 code, 70% included a measles diagnosis code (B05) recorded as a primary or secondary reason for admission, suggesting reasonable concordance. However, measles coding may have been missing for some admissions if laboratory results became available after discharge. Nonetheless, ICD-10 coding does not require laboratory confirmation and may therefore overestimate or underestimate measles-related hospitalisations, highlighting the value of linking surveillance and hospital data. Hospital admission may overestimate disease severity, as the short median LOS suggests that some admissions, particularly among younger children, were precautionary. A small number of hospital-acquired measles cases were included; for which admission and LOS are not reliable proxies for severity. Stratification by age resulted in small numbers in some groups, leading to wide confidence intervals, requiring cautious interpretation.

Although measles virus sequencing is routinely undertaken in England and informs outbreak investigations, sequence-level analyses were not included in this paper. As multiple sequence variants may circulate within a single genotype, this limited differentiation of importations from ongoing transmission and reduced precision in defining transmission chains.

Finally, vaccination records within IIS are incomplete, with data quality declining in older children and young adults. As a result, some adolescents and adults may have been misclassified as unvaccinated.

## Conclusion

This outbreak highlights a shift in measles epidemiology in the UK, from outbreaks concentrated in key undervaccinated communities to broader transmission in the population driven by declining MMR coverage. This highlights the urgent need to restore and sustain ≥ 95% coverage for both MMR (now MMRV) doses. This analysis underscores the importance of timely surveillance and the opportunity it provides for intervention through targeted catch-up vaccination campaigns in hot spots. With the growing availability of data platforms, we can further refine these targeted catch-up efforts using data such as school-level MMR coverage, helping to identify pockets of at-risk populations. Additionally, this study serves as a baseline for comparing outbreaks across the MMR and MMRV eras, enabling assessment of this most recent change to the programme.

## Data Availability

This work is carried out under Regulation 3 of The Health Service (Control of Patient Information; Secretary of State for Health, 2002) using patient identification information without individual patient consent as part of the UKHSA legal requirement for public health surveillance and monitoring of vaccines. As such, authors cannot make the underlying dataset publicly available for ethical and legal reasons. Applications for relevant anonymised data should be submitted to the UKHSA Office for Data Release at https://www.gov.uk/government/publications/accessing-ukhsa-protected-data.

## Supplementary Data

167000 Supplementary Material
