# Supplementary material for: Measles resurgence in England: challenges in maintaining elimination, 2023 to 2024
Source: Euro Surveill. 2026 Nov 12;31(45):2600124. doi: 10.2807/1560-7917.ES.2026.31.45.2600124 (PMC13768765; doi:10.2807/1560-7917.ES.2026.31.45.2600124)
Supplement: Supplementary file 1 [file 26-00124_SALIBA_Supplement.pdf]

Supplementary Material

Supplementary Figure S1. Enhanced measles surveillance data flow in England, 2023–2024*.

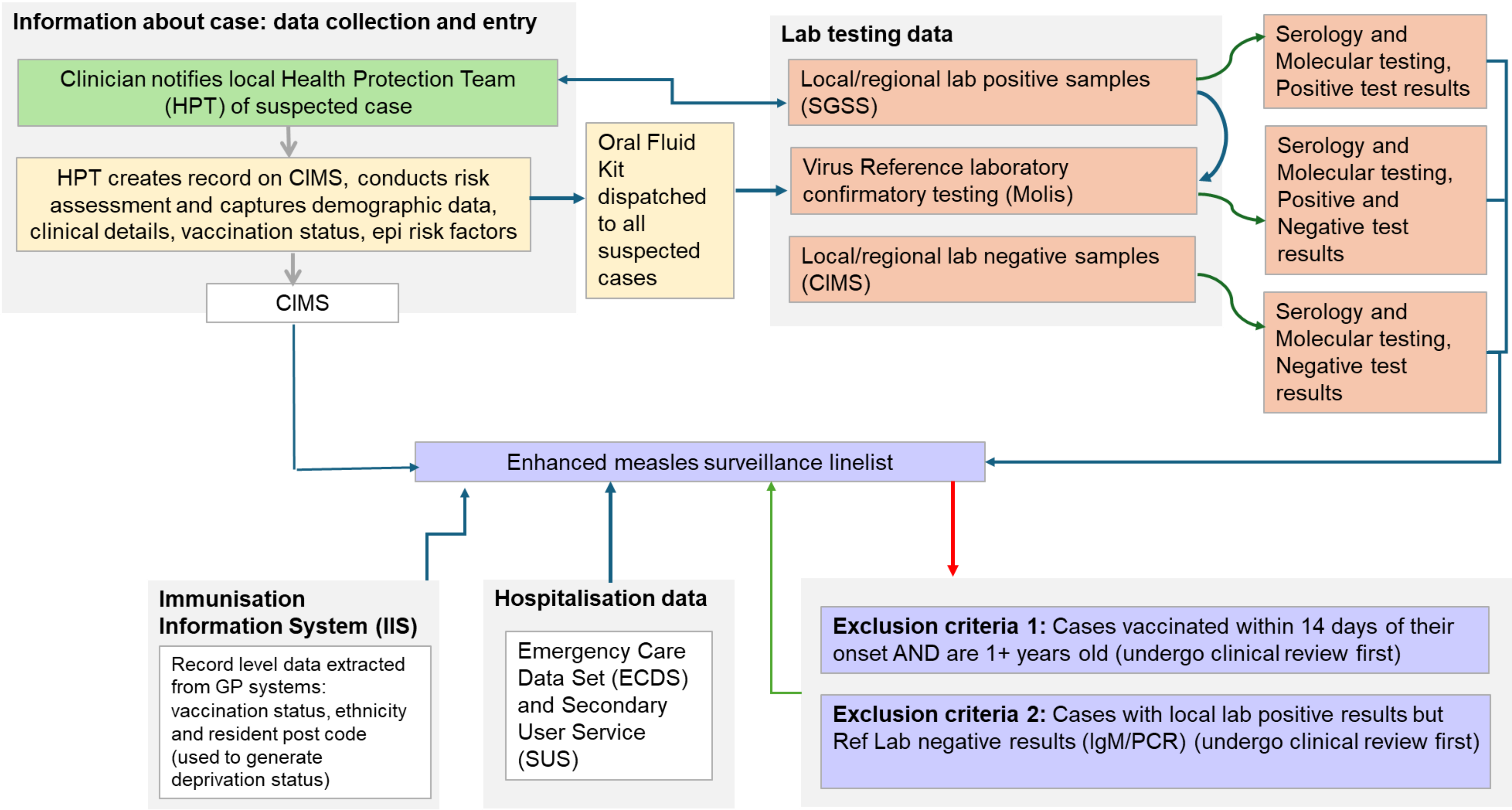

* The flow diagram illustrates the pathway from suspected measles case identification to inclusion in the enhanced national surveillance linelist. It outlines the laboratory surveillance sources and associated diagnostic tests, alongside the data sources used to supplement case vaccination and demographic information.

Disclaimer: This supplementary material is hosted by Eurosurveillance as supporting information alongside the article “Measles resurgence in England: challenges in maintaining elimination, 2023 to 2024”, on behalf of the authors, who remain responsible for the accuracy and appropriateness of the content. The same standards for ethics, copyright, attributions and permissions as for the article apply. Supplements are not edited by Eurosurveillance and the journal is not responsible for the maintenance of any links or email addresses provided therein.
